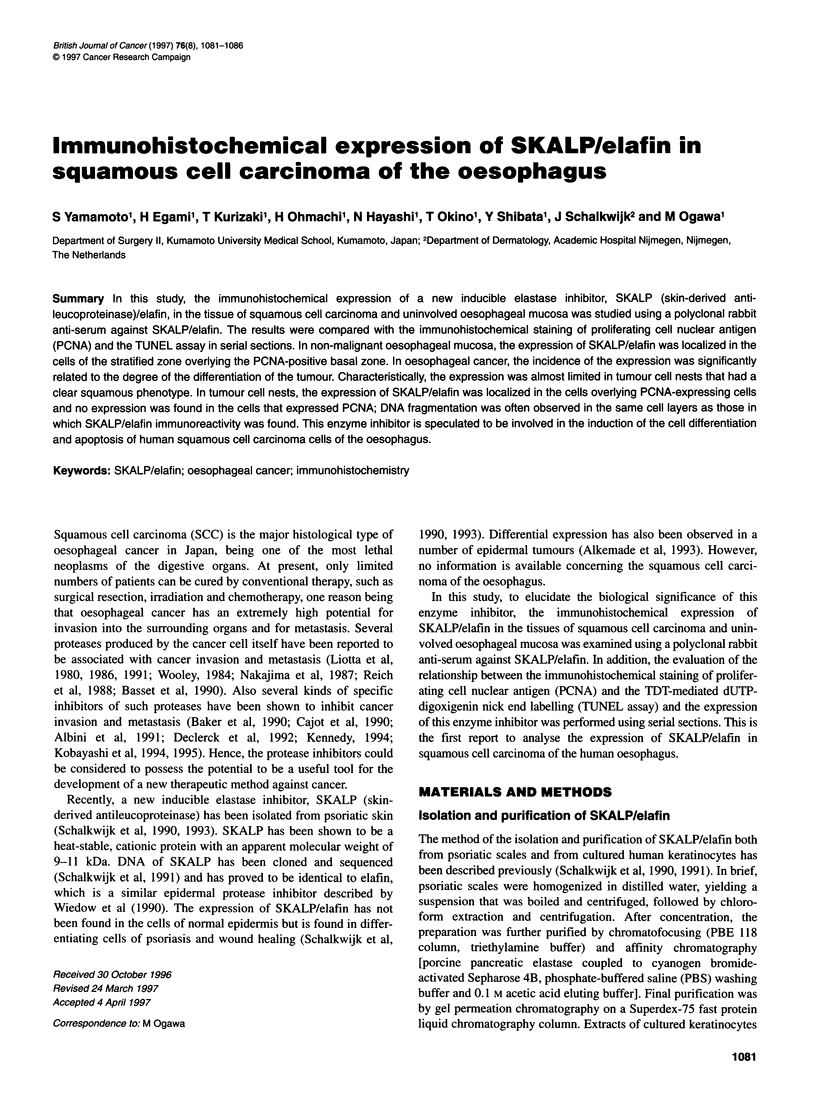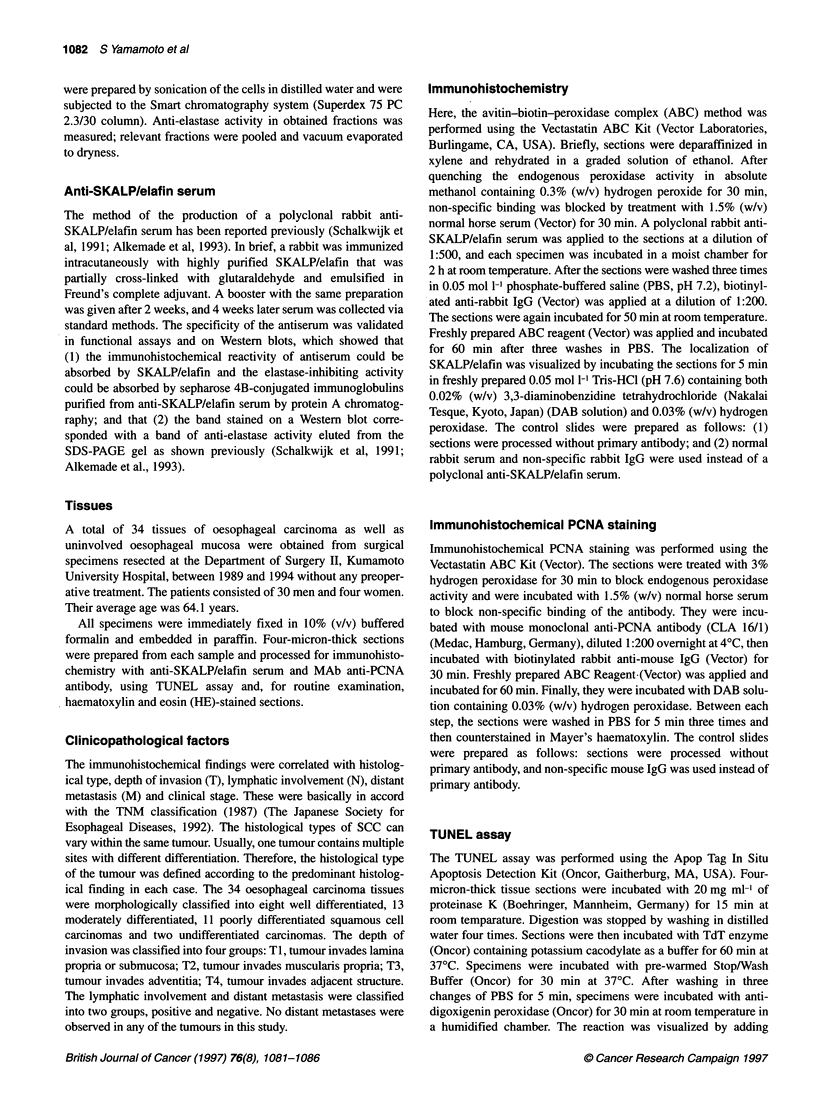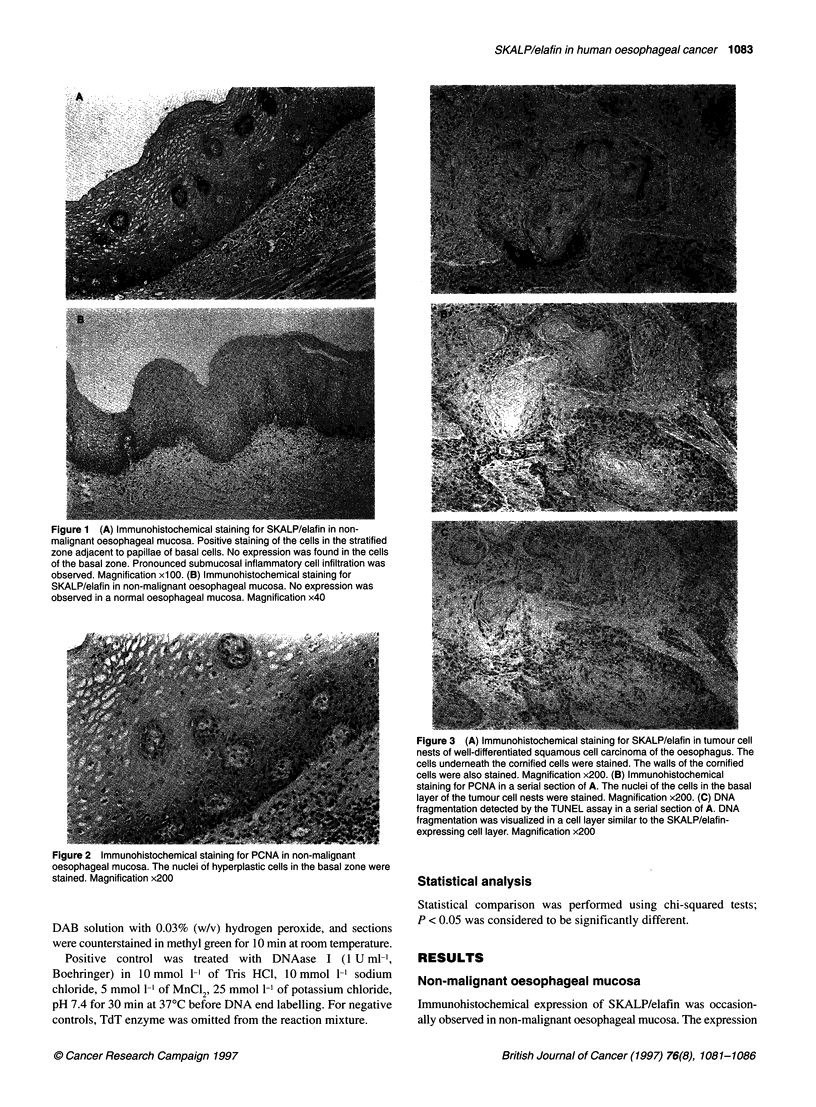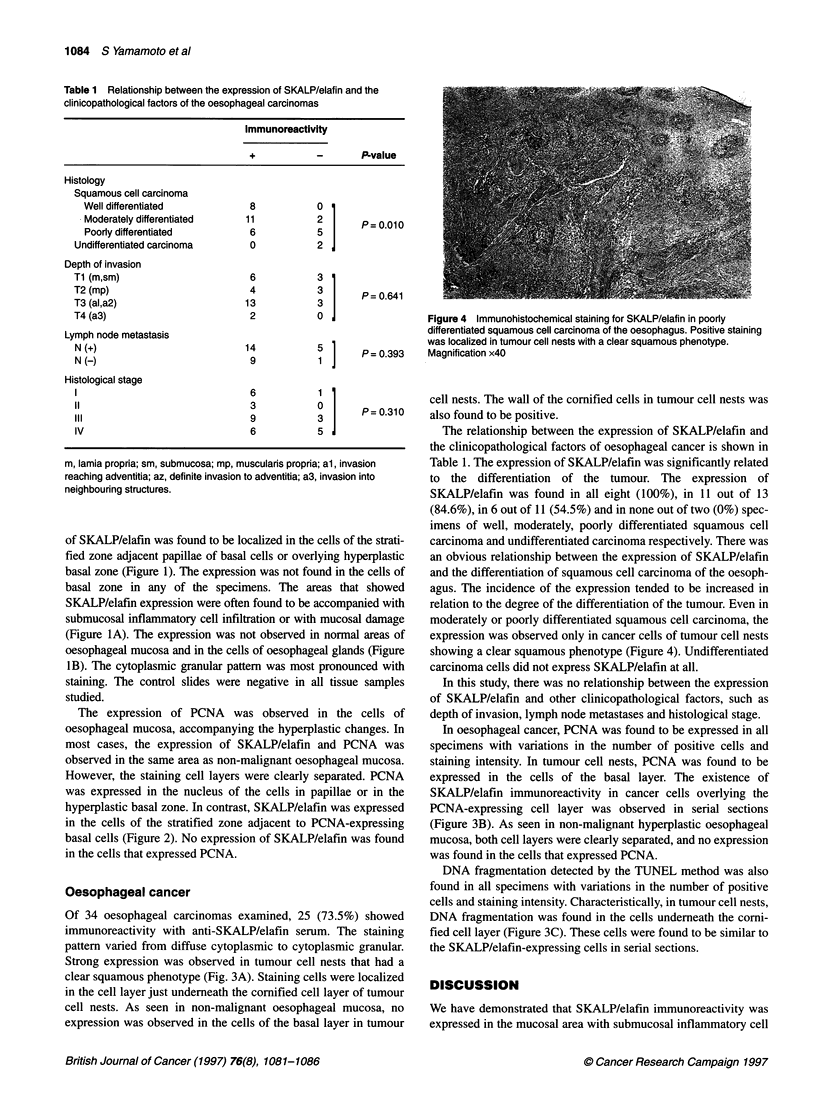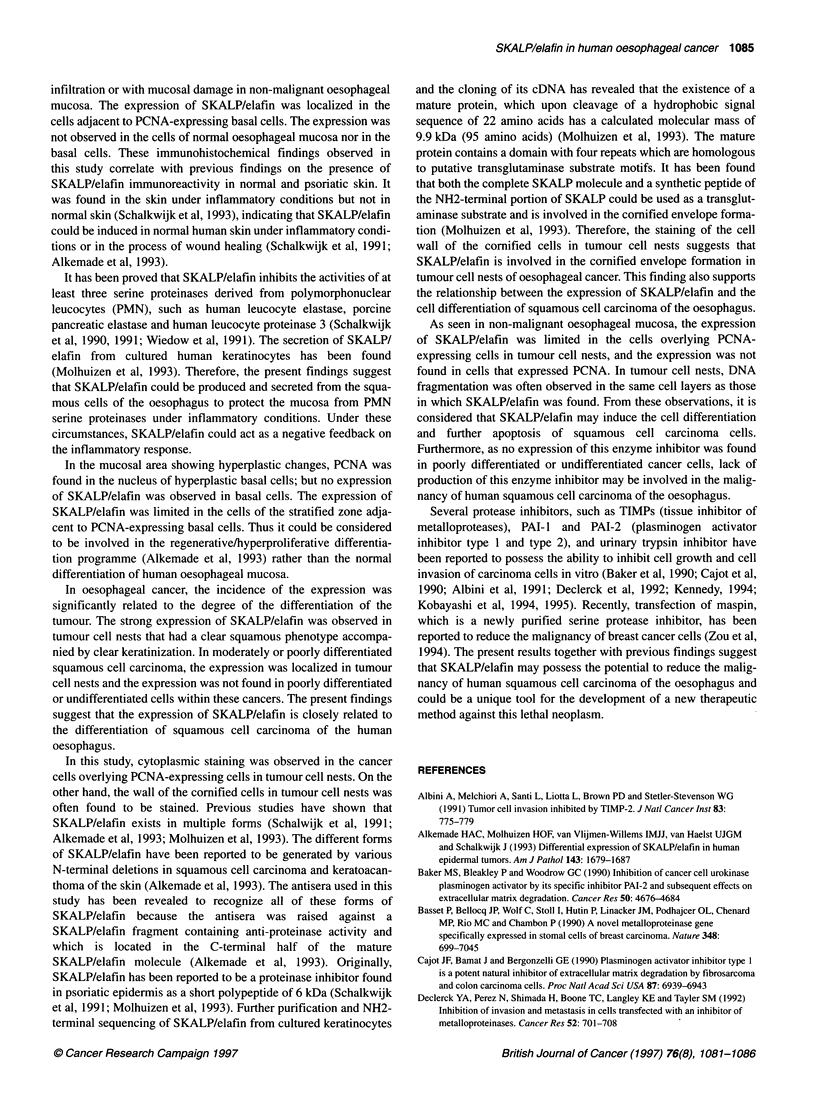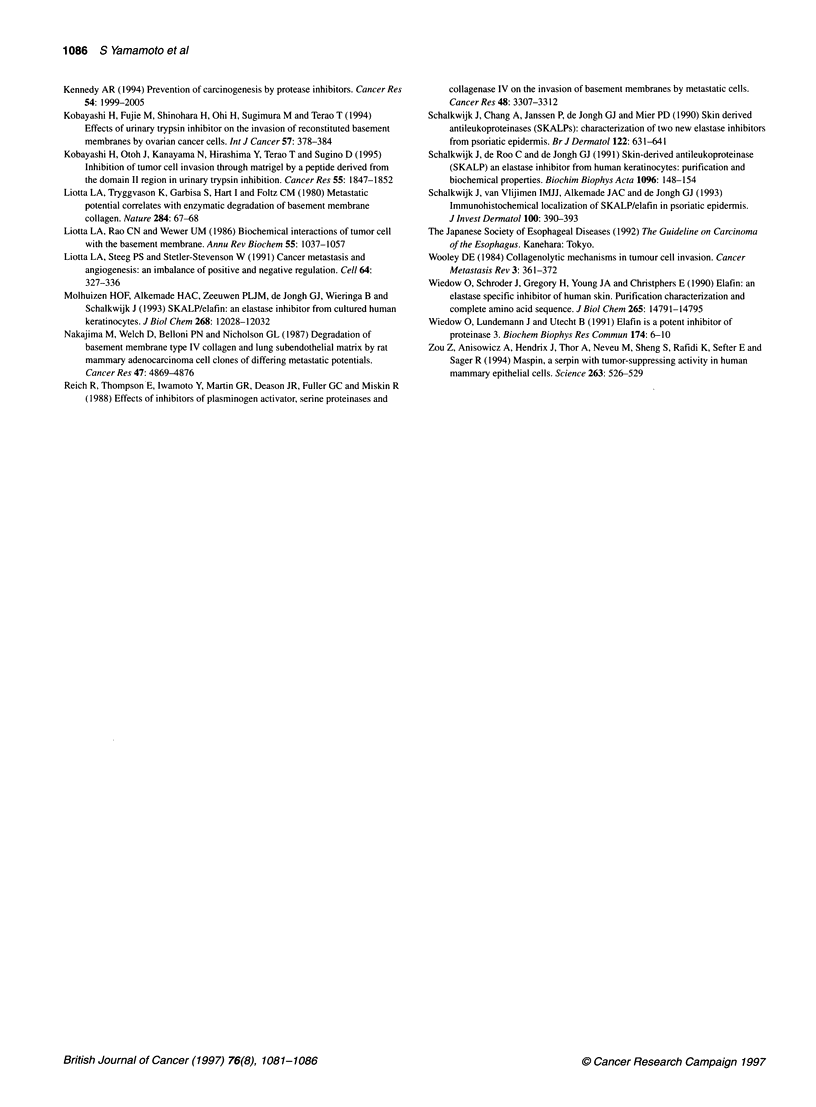# Immunohistochemical expression of SKALP/elafin in squamous cell carcinoma of the oesophagus.

**DOI:** 10.1038/bjc.1997.511

**Published:** 1997

**Authors:** S. Yamamoto, H. Egami, T. Kurizaki, H. Ohmachi, N. Hayashi, T. Okino, Y. Shibata, J. Schalkwijk, M. Ogawa

**Affiliations:** Department of Surgery II, Kumamoto University Medical School, Japan.

## Abstract

**Images:**


British Journal of Cancer (1997) 76(8), 1081–1086
© 1997 Cancer Research Campaign

### Immunohistochemical expression of SKALP/elafin in squamous cell carcinoma of the oesophagus

S Yamamoto[1], H Egami[1], T Kurizaki[1], H Ohmachi[1], N Hayashi[1], T Okino[1], Y Shibata[1], J Schalkwijk[2] and M Ogawa[1]

Department of Surgery II, Kumamoto University Medical School, Kumamoto, Japan; [2]Department of Dermatology, Academic Hospital Nijmegen, Nijmegen, The Netherlands

**Summary** In this study, the immunohistochemical expression of a new inducible elastase inhibitor, SKALP (skin-derived anti-leucoproteinase)/elafin, in the tissue of squamous cell carcinoma and uninvolved oesophageal mucosa was studied using a polyclonal rabbit anti-serum against SKALP/elafin. The results were compared with the immunohistochemical staining of proliferating cell nuclear antigen (PCNA) and the TUNEL assay in serial sections. In non-malignant oesophageal mucosa, the expression of SKALP/elafin was localized in the cells of the stratified zone overlying the PCNA-positive basal zone. In oesophageal cancer, the incidence of the expression was significantly related to the degree of the differentiation of the tumour. Characteristically, the expression was almost limited in tumour cell nests that had a clear squamous phenotype. In tumour cell nests, the expression of SKALP/elafin was localized in the cells overlying PCNA-expressing cells and no expression was found in the cells that expressed PCNA; DNA fragmentation was often observed in the same cell layers as those in which SKALP/elafin immunoreactivity was found. This enzyme inhibitor is speculated to be involved in the induction of the cell differentiation and apoptosis of human squamous cell carcinoma cells of the oesophagus.

**Keywords:** SKALP/elafin; oesophageal cancer; immunohistochemistry

Squamous cell carcinoma (SCC) is the major histological type of oesophageal cancer in Japan, being one of the most lethal neoplasms of the digestive organs. At present, only limited numbers of patients can be cured by conventional therapy, such as surgical resection, irradiation and chemotherapy, one reason being that oesophageal cancer has an extremely high potential for invasion into the surrounding organs and for metastasis. Several proteases produced by the cancer cell itself have been reported to be associated with cancer invasion and metastasis (Liotta et al, 1980, 1986, 1991; Wooley, 1984; Nakajima et al, 1987; Reich et al, 1988; Basset et al, 1990). Also several kinds of specific inhibitors of such proteases have been shown to inhibit cancer invasion and metastasis (Baker et al, 1990; Cajot et al, 1990; Albini et al, 1991; Declerck et al, 1992; Kennedy, 1994; Kobayashi et al, 1994, 1995). Hence, the protease inhibitors could be considered to possess the potential to be a useful tool for the development of a new therapeutic method against cancer.

Recently, a new inducible elastase inhibitor, SKALP (skin-derived antileucoproteinase) has been isolated from psoriatic skin (Schalkwijk et al, 1990, 1993). SKALP has been shown to be a heat-stable, cationic protein with an apparent molecular weight of 9–11 kDa. DNA of SKALP has been cloned and sequenced (Schalkwijk et al, 1991) and has proved to be identical to elafin, which is a similar epidermal protease inhibitor described by Wiedow et al (1990). The expression of SKALP/elafin has not been found in the cells of normal epidermis but is found in differentiating cells of psoriasis and wound healing (Schalkwijk et al, 1990, 1993). Differential expression has also been observed in a number of epidermal tumours (Alkemade et al, 1993). However, no information is available concerning the squamous cell carcinoma of the oesophagus.

In this study, to elucidate the biological significance of this enzyme inhibitor, the immunohistochemical expression of SKALP/elafin in the tissues of squamous cell carcinoma and uninvolved oesophageal mucosa was examined using a polyclonal rabbit anti-serum against SKALP/elafin. In addition, the evaluation of the relationship between the immunohistochemical staining of proliferating cell nuclear antigen (PCNA) and the TDT-mediated dUTP-digoxigenin nick end labelling (TUNEL assay) and the expression of this enzyme inhibitor was performed using serial sections. This is the first report to analyse the expression of SKALP/elafin in squamous cell carcinoma of the human oesophagus.

*Received 30 October 1996*
*Revised 24 March 1997*
*Accepted 4 April 1997*

*Correspondence to:* M Ogawa

### MATERIALS AND METHODS

### Isolation and purification of SKALP/elafin

The method of the isolation and purification of SKALP/elafin both from psoriatic scales and from cultured human keratinocytes has been described previously (Schalkwijk et al, 1990, 1991). In brief, psoriatic scales were homogenized in distilled water, yielding a suspension that was boiled and centrifuged, followed by chloroform extraction and centrifugation. After concentration, the preparation was further purified by chromatofocusing (PBE 118 column, triethylamine buffer) and affinity chromatography [porcine pancreatic elastase coupled to cyanogen bromide-activated Sepharose 4B, phosphate-buffered saline (PBS) washing buffer and 0.1 M acetic acid eluting buffer]. Final purification was by gel permeation chromatography on a Superdex-75 fast protein liquid chromatography column. Extracts of cultured keratinocytes were prepared by sonication of the cells in distilled water and were subjected to the Smart chromatography system (Superdex 75 PC 2.3/30 column). Anti-elastase activity in obtained fractions was measured; relevant fractions were pooled and vacuum evaporated to dryness.

### Anti-SKALP/elafin serum

The method of the production of a polyclonal rabbit anti-SKALP/elafin serum has been reported previously (Schalkwijk et al, 1991; Alkemade et al, 1993). In brief, a rabbit was immunized intracutaneously with highly purified SKALP/elafin that was partially cross-linked with glutaraldehyde and emulsified in Freund's complete adjuvant. A booster with the same preparation was given after 2 weeks, and 4 weeks later serum was collected via standard methods. The specificity of the antiserum was validated in functional assays and on Western blots, which showed that (1) the immunohistochemical reactivity of antiserum could be absorbed by SKALP/elafin and the elastase-inhibiting activity could be absorbed by sepharose 4B-conjugated immunoglobulins purified from anti-SKALP/elafin serum by protein A chromatography; and that (2) the band stained on a Western blot corresponded with a band of anti-elastase activity eluted from the SDS-PAGE gel as shown previously (Schalkwijk et al, 1991; Alkemade et al., 1993).

### Tissues

A total of 34 tissues of oesophageal carcinoma as well as uninvolved oesophageal mucosa were obtained from surgical specimens resected at the Department of Surgery II, Kumamoto University Hospital, between 1989 and 1994 without any preoperative treatment. The patients consisted of 30 men and four women. Their average age was 64.1 years.

All specimens were immediately fixed in 10% (v/v) buffered formalin and embedded in paraffin. Four-micron-thick sections were prepared from each sample and processed for immunohistochemistry with anti-SKALP/elafin serum and MAb anti-PCNA antibody, using TUNEL assay and, for routine examination, haematoxylin and eosin (HE)-stained sections.

### Clinicopathological factors

The immunohistochemical findings were correlated with histological type, depth of invasion (T), lymphatic involvement (N), distant metastasis (M) and clinical stage. These were basically in accord with the TNM classification (1987) (The Japanese Society for Esophageal Diseases, 1992). The histological types of SCC can vary within the same tumour. Usually, one tumour contains multiple sites with different differentiation. Therefore, the histological type of the tumour was defined according to the predominant histological finding in each case. The 34 oesophageal carcinoma tissues were morphologically classified into eight well differentiated, 13 moderately differentiated, 11 poorly differentiated squamous cell carcinomas and two undifferentiated carcinomas. The depth of invasion was classified into four groups: T1, tumour invades lamina propria or submucosa; T2, tumour invades muscularis propria; T3, tumour invades adventitia; T4, tumour invades adjacent structure. The lymphatic involvement and distant metastasis were classified into two groups, positive and negative. No distant metastases were observed in any of the tumours in this study.

### Immunohistochemistry

Here, the avitin–biotin–peroxidase complex (ABC) method was performed using the Vectastatin ABC Kit (Vector Laboratories, Burlingame, CA, USA). Briefly, sections were deparaffinized in xylene and rehydrated in a graded solution of ethanol. After quenching the endogenous peroxidase activity in absolute methanol containing 0.3% (w/v) hydrogen peroxide for 30 min, non-specific binding was blocked by treatment with 1.5% (w/v) normal horse serum (Vector) for 30 min. A polyclonal rabbit anti-SKALP/elafin serum was applied to the sections at a dilution of 1:500, and each specimen was incubated in a moist chamber for 2 h at room temperature. After the sections were washed three times in 0.05 mol $l^{-1}$ phosphate-buffered saline (PBS, pH 7.2), biotinylated anti-rabbit IgG (Vector) was applied at a dilution of 1:200. The sections were again incubated for 50 min at room temperature. Freshly prepared ABC reagent (Vector) was applied and incubated for 60 min after three washes in PBS. The localization of SKALP/elafin was visualized by incubating the sections for 5 min in freshly prepared 0.05 mol $l^{-1}$ Tris-HCl (pH 7.6) containing both 0.02% (w/v) 3,3-diaminobenzidine tetrahydrochloride (Nakalai Tesque, Kyoto, Japan) (DAB solution) and 0.03% (w/v) hydrogen peroxidase. The control slides were prepared as follows: (1) sections were processed without primary antibody; and (2) normal rabbit serum and non-specific rabbit IgG were used instead of a polyclonal anti-SKALP/elafin serum.

### Immunohistochemical PCNA staining

Immunohistochemical PCNA staining was performed using the Vectastatin ABC Kit (Vector). The sections were treated with 3% hydrogen peroxidase for 30 min to block endogenous peroxidase activity and were incubated with 1.5% (w/v) normal horse serum to block non-specific binding of the antibody. They were incubated with mouse monoclonal anti-PCNA antibody (CLA 16/1) (Medac, Hamburg, Germany), diluted 1:200 overnight at 4°C, then incubated with biotinylated rabbit anti-mouse IgG (Vector) for 30 min. Freshly prepared ABC Reagent (Vector) was applied and incubated for 60 min. Finally, they were incubated with DAB solution containing 0.03% (w/v) hydrogen peroxidase. Between each step, the sections were washed in PBS for 5 min three times and then counterstained in Mayer's haematoxylin. The control slides were prepared as follows: sections were processed without primary antibody, and non-specific mouse IgG was used instead of primary antibody.

### TUNEL assay

The TUNEL assay was performed using the Apop Tag In Situ Apoptosis Detection Kit (Oncor, Gaitherburg, MA, USA). Four-micron-thick tissue sections were incubated with 20 mg $ml^{-1}$ of proteinase K (Boehringer, Mannheim, Germany) for 15 min at room temparature. Digestion was stopped by washing in distilled water four times. Sections were then incubated with TdT enzyme (Oncor) containing potassium cacodylate as a buffer for 60 min at 37°C. Specimens were incubated with pre-warmed Stop/Wash Buffer (Oncor) for 30 min at 37°C. After washing in three changes of PBS for 5 min, specimens were incubated with anti-digoxigenin peroxidase (Oncor) for 30 min at room temperature in a humidified chamber. The reaction was visualized by adding

© Cancer Research Campaign 1997

**Figure 1** (**A**) Immunohistochemical staining for SKALP/elafin in non-malignant oesophageal mucosa. Positive staining of the cells in the stratified zone adjacent to papillae of basal cells. No expression was found in the cells of the basal zone. Pronounced submucosal inflammatory cell infiltration was observed. Magnification ×100. (**B**) Immunohistochemical staining for SKALP/elafin in non-malignant oesophageal mucosa. No expression was observed in a normal oesophageal mucosa. Magnification ×40

**Figure 2** Immunohistochemical staining for PCNA in non-malignant oesophageal mucosa. The nuclei of hyperplastic cells in the basal zone were stained. Magnification ×200

DAB solution with 0.03% (w/v) hydrogen peroxide, and sections were counterstained in methyl green for 10 min at room temperature.

Positive control was treated with DNAase I (1 U $ml^{-1}$, Boehringer) in 10 mmol $l^{-1}$ of Tris HCl, 10 mmol $l^{-1}$ sodium chloride, 5 mmol $l^{-1}$ of $MnCl_2$, 25 mmol $l^{-1}$ of potassium chloride, pH 7.4 for 30 min at 37°C before DNA end labelling. For negative controls, TdT enzyme was omitted from the reaction mixture.

**Figure 3** (**A**) Immunohistochemical staining for SKALP/elafin in tumour cell nests of well-differentiated squamous cell carcinoma of the oesophagus. The cells underneath the cornified cells were stained. The walls of the cornified cells were also stained. Magnification ×200. (**B**) Immunohistochemical staining for PCNA in a serial section of **A**. The nuclei of the cells in the basal layer of the tumour cell nests were stained. Magnification ×200. (**C**) DNA fragmentation detected by the TUNEL assay in a serial section of **A**. DNA fragmentation was visualized in a cell layer similar to the SKALP/elafin-expressing cell layer. Magnification ×200

### Statistical analysis

Statistical comparison was performed using chi-squared tests; $P < 0.05$ was considered to be significantly different.

### RESULTS

### Non-malignant oesophageal mucosa

Immunohistochemical expression of SKALP/elafin was occasionally observed in non-malignant oesophageal mucosa. The expression

© Cancer Research Campaign 1997

**Table 1** Relationship between the expression of SKALP/elafin and the clinicopathological factors of the oesophageal carcinomas

| | Immunoreactivity | | |
|---|---|---|---|
| | + | – | *P*-value |
| Histology | | | |
| Squamous cell carcinoma | | | |
| Well differentiated | 8 | 0 | *P* = 0.010 |
| Moderately differentiated | 11 | 2 | |
| Poorly differentiated | 6 | 5 | |
| Undifferentiated carcinoma | 0 | 2 | |
| Depth of invasion | | | |
| T1 (m,sm) | 6 | 3 | *P* = 0.641 |
| T2 (mp) | 4 | 3 | |
| T3 (al,a2) | 13 | 3 | |
| T4 (a3) | 2 | 0 | |
| Lymph node metastasis | | | |
| N (+) | 14 | 5 | *P* = 0.393 |
| N (–) | 9 | 1 | |
| Histological stage | | | |
| I | 6 | 1 | *P* = 0.310 |
| II | 3 | 0 | |
| III | 9 | 3 | |
| IV | 6 | 5 | |

m, lamia propria; sm, submucosa; mp, muscularis propria; a1, invasion reaching adventitia; az, definite invasion to adventitia; a3, invasion into neighbouring structures.

of SKALP/elafin was found to be localized in the cells of the stratified zone adjacent papillae of basal cells or overlying hyperplastic basal zone (Figure 1). The expression was not found in the cells of basal zone in any of the specimens. The areas that showed SKALP/elafin expression were often found to be accompanied with submucosal inflammatory cell infiltration or with mucosal damage (Figure 1A). The expression was not observed in normal areas of oesophageal mucosa and in the cells of oesophageal glands (Figure 1B). The cytoplasmic granular pattern was most pronounced with staining. The control slides were negative in all tissue samples studied.

The expression of PCNA was observed in the cells of oesophageal mucosa, accompanying the hyperplastic changes. In most cases, the expression of SKALP/elafin and PCNA was observed in the same area as non-malignant oesophageal mucosa. However, the staining cell layers were clearly separated. PCNA was expressed in the nucleus of the cells in papillae or in the hyperplastic basal zone. In contrast, SKALP/elafin was expressed in the cells of the stratified zone adjacent to PCNA-expressing basal cells (Figure 2). No expression of SKALP/elafin was found in the cells that expressed PCNA.

### Oesophageal cancer

Of 34 oesophageal carcinomas examined, 25 (73.5%) showed immunoreactivity with anti-SKALP/elafin serum. The staining pattern varied from diffuse cytoplasmic to cytoplasmic granular. Strong expression was observed in tumour cell nests that had a clear squamous phenotype (Fig. 3A). Staining cells were localized in the cell layer just underneath the cornified cell layer of tumour cell nests. As seen in non-malignant oesophageal mucosa, no expression was observed in the cells of the basal layer in tumour cell nests. The wall of the cornified cells in tumour cell nests was also found to be positive.

**Figure 4** Immunohistochemical staining for SKALP/elafin in poorly differentiated squamous cell carcinoma of the oesophagus. Positive staining was localized in tumour cell nests with a clear squamous phenotype. Magnification ×40

The relationship between the expression of SKALP/elafin and the clinicopathological factors of oesophageal cancer is shown in Table 1. The expression of SKALP/elafin was significantly related to the differentiation of the tumour. The expression of SKALP/elafin was found in all eight (100%), in 11 out of 13 (84.6%), in 6 out of 11 (54.5%) and in none out of two (0%) specimens of well, moderately, poorly differentiated squamous cell carcinoma and undifferentiated carcinoma respectively. There was an obvious relationship between the expression of SKALP/elafin and the differentiation of squamous cell carcinoma of the oesophagus. The incidence of the expression tended to be increased in relation to the degree of the differentiation of the tumour. Even in moderately or poorly differentiated squamous cell carcinoma, the expression was observed only in cancer cells of tumour cell nests showing a clear squamous phenotype (Figure 4). Undifferentiated carcinoma cells did not express SKALP/elafin at all.

In this study, there was no relationship between the expression of SKALP/elafin and other clinicopathological factors, such as depth of invasion, lymph node metastases and histological stage.

In oesophageal cancer, PCNA was found to be expressed in all specimens with variations in the number of positive cells and staining intensity. In tumour cell nests, PCNA was found to be expressed in the cells of the basal layer. The existence of SKALP/elafin immunoreactivity in cancer cells overlying the PCNA-expressing cell layer was observed in serial sections (Figure 3B). As seen in non-malignant hyperplastic oesophageal mucosa, both cell layers were clearly separated, and no expression was found in the cells that expressed PCNA.

DNA fragmentation detected by the TUNEL method was also found in all specimens with variations in the number of positive cells and staining intensity. Characteristically, in tumour cell nests, DNA fragmentation was found in the cells underneath the cornified cell layer (Figure 3C). These cells were found to be similar to the SKALP/elafin-expressing cells in serial sections.

### DISCUSSION

We have demonstrated that SKALP/elafin immunoreactivity was expressed in the mucosal area with submucosal inflammatory cell

© Cancer Research Campaign 1997

infiltration or with mucosal damage in non-malignant oesophageal mucosa. The expression of SKALP/elafin was localized in the cells adjacent to PCNA-expressing basal cells. The expression was not observed in the cells of normal oesophageal mucosa nor in the basal cells. These immunohistochemical findings observed in this study correlate with previous findings on the presence of SKALP/elafin immunoreactivity in normal and psoriatic skin. It was found in the skin under inflammatory conditions but not in normal skin (Schalkwijk et al, 1993), indicating that SKALP/elafin could be induced in normal human skin under inflammatory conditions or in the process of wound healing (Schalkwijk et al, 1991; Alkemade et al, 1993).

It has been proved that SKALP/elafin inhibits the activities of at least three serine proteinases derived from polymorphonuclear leucocytes (PMN), such as human leucocyte elastase, porcine pancreatic elastase and human leucocyte proteinase 3 (Schalkwijk et al, 1990, 1991; Wiedow et al, 1991). The secretion of SKALP/elafin from cultured human keratinocytes has been found (Molhuizen et al, 1993). Therefore, the present findings suggest that SKALP/elafin could be produced and secreted from the squamous cells of the oesophagus to protect the mucosa from PMN serine proteinases under inflammatory conditions. Under these circumstances, SKALP/elafin could act as a negative feedback on the inflammatory response.

In the mucosal area showing hyperplastic changes, PCNA was found in the nucleus of hyperplastic basal cells; but no expression of SKALP/elafin was observed in basal cells. The expression of SKALP/elafin was limited in the cells of the stratified zone adjacent to PCNA-expressing basal cells. Thus it could be considered to be involved in the regenerative/hyperproliferative differentiation programme (Alkemade et al, 1993) rather than the normal differentiation of human oesophageal mucosa.

In oesophageal cancer, the incidence of the expression was significantly related to the degree of the differentiation of the tumour. The strong expression of SKALP/elafin was observed in tumour cell nests that had a clear squamous phenotype accompanied by clear keratinization. In moderately or poorly differentiated squamous cell carcinoma, the expression was localized in tumour cell nests and the expression was not found in poorly differentiated or undifferentiated cells within these cancers. The present findings suggest that the expression of SKALP/elafin is closely related to the differentiation of squamous cell carcinoma of the human oesophagus.

In this study, cytoplasmic staining was observed in the cancer cells overlying PCNA-expressing cells in tumour cell nests. On the other hand, the wall of the cornified cells in tumour cell nests was often found to be stained. Previous studies have shown that SKALP/elafin exists in multiple forms (Schalwijk et al, 1991; Alkemade et al, 1993; Molhuizen et al, 1993). The different forms of SKALP/elafin have been reported to be generated by various N-terminal deletions in squamous cell carcinoma and keratoacanthoma of the skin (Alkemade et al, 1993). The antisera used in this study has been revealed to recognize all of these forms of SKALP/elafin because the antisera was raised against a SKALP/elafin fragment containing anti-proteinase activity and which is located in the C-terminal half of the mature SKALP/elafin molecule (Alkemade et al, 1993). Originally, SKALP/elafin has been reported to be a proteinase inhibitor found in psoriatic epidermis as a short polypeptide of 6 kDa (Schalkwijk et al, 1991; Molhuizen et al, 1993). Further purification and NH2-terminal sequencing of SKALP/elafin from cultured keratinocytes and the cloning of its cDNA has revealed that the existence of a mature protein, which upon cleavage of a hydrophobic signal sequence of 22 amino acids has a calculated molecular mass of 9.9 kDa (95 amino acids) (Molhuizen et al, 1993). The mature protein contains a domain with four repeats which are homologous to putative transglutaminase substrate motifs. It has been found that both the complete SKALP molecule and a synthetic peptide of the NH2-terminal portion of SKALP could be used as a transglutaminase substrate and is involved in the cornified envelope formation (Molhuizen et al, 1993). Therefore, the staining of the cell wall of the cornified cells in tumour cell nests suggests that SKALP/elafin is involved in the cornified envelope formation in tumour cell nests of oesophageal cancer. This finding also supports the relationship between the expression of SKALP/elafin and the cell differentiation of squamous cell carcinoma of the oesophagus.

As seen in non-malignant oesophageal mucosa, the expression of SKALP/elafin was limited in the cells overlying PCNA-expressing cells in tumour cell nests, and the expression was not found in cells that expressed PCNA. In tumour cell nests, DNA fragmentation was often observed in the same cell layers as those in which SKALP/elafin was found. From these observations, it is considered that SKALP/elafin may induce the cell differentiation and further apoptosis of squamous cell carcinoma cells. Furthermore, as no expression of this enzyme inhibitor was found in poorly differentiated or undifferentiated cancer cells, lack of production of this enzyme inhibitor may be involved in the malignancy of human squamous cell carcinoma of the oesophagus.

Several protease inhibitors, such as TIMPs (tissue inhibitor of metalloproteases), PAI-1 and PAI-2 (plasminogen activator inhibitor type 1 and type 2), and urinary trypsin inhibitor have been reported to possess the ability to inhibit cell growth and cell invasion of carcinoma cells in vitro (Baker et al, 1990; Cajot et al, 1990; Albini et al, 1991; Declerck et al, 1992; Kennedy, 1994; Kobayashi et al, 1994, 1995). Recently, transfection of maspin, which is a newly purified serine protease inhibitor, has been reported to reduce the malignancy of breast cancer cells (Zou et al, 1994). The present results together with previous findings suggest that SKALP/elafin may possess the potential to reduce the malignancy of human squamous cell carcinoma of the oesophagus and could be a unique tool for the development of a new therapeutic method against this lethal neoplasm.

### REFERENCES

Albini A, Melchiori A, Santi L, Liotta L, Brown PD and Stetler-Stevenson WG (1991) Tumor cell invasion inhibited by TIMP-2. *J Natl Cancer Inst* **83**: 775–779

Alkemade HAC, Molhuizen HOF, van Vlijmen-Willems IMJJ, van Haelst UJGM and Schalkwijk J (1993) Differential expression of SKALP/elafin in human epidermal tumors. *Am J Pathol* **143**: 1679–1687

Baker MS, Bleakley P and Woodrow GC (1990) Inhibition of cancer cell urokinase plasminogen activator by its specific inhibitor PAI-2 and subsequent effects on extracellular matrix degradation. *Cancer Res* **50**: 4676–4684

Basset P, Bellocq JP, Wolf C, Stoll I, Hutin P, Linacker JM, Podhajcer OL, Chenard MP, Rio MC and Chambon P (1990) A novel metalloproteinase gene specifically expressed in stomal cells of breast carcinoma. *Nature* **348**: 699–7045

Cajot JF, Bamat J and Bergonzelli GE (1990) Plasminogen activator inhibitor type 1 is a potent natural inhibitor of extracellular matrix degradation by fibrosarcoma and colon carcinoma cells. *Proc Natl Acad Sci USA* **87**: 6939–6943

Declerck YA, Perez N, Shimada H, Boone TC, Langley KE and Tayler SM (1992) Inhibition of invasion and metastasis in cells transfected with an inhibitor of metalloproteinases. *Cancer Res* **52**: 701–708

© Cancer Research Campaign 1997

Kennedy AR (1994) Prevention of carcinogenesis by protease inhibitors. *Cancer Res* **54**: 1999–2005

Kobayashi H, Fujie M, Shinohara H, Ohi H, Sugimura M and Terao T (1994) Effects of urinary trypsin inhibitor on the invasion of reconstituted basement membranes by ovarian cancer cells. *Int J Cancer* **57**: 378–384

Kobayashi H, Otoh J, Kanayama N, Hirashima Y, Terao T and Sugino D (1995) Inhibition of tumor cell invasion through matrigel by a peptide derived from the domain II region in urinary trypsin inhibition. *Cancer Res* **55**: 1847–1852

Liotta LA, Tryggvason K, Garbisa S, Hart I and Foltz CM (1980) Metastatic potential correlates with enzymatic degradation of basement membrane collagen. *Nature* **284**: 67–68

Liotta LA, Rao CN and Wewer UM (1986) Biochemical interactions of tumor cell with the basement membrane. *Annu Rev Biochem* **55**: 1037–1057

Liotta LA, Steeg PS and Stetler-Stevenson W (1991) Cancer metastasis and angiogenesis: an imbalance of positive and negative regulation. *Cell* **64**: 327–336

Molhuizen HOF, Alkemade HAC, Zeeuwen PLJM, de Jongh GJ, Wieringa B and Schalkwijk J (1993) SKALP/elafin: an elastase inhibitor from cultured human keratinocytes. *J Biol Chem* **268**: 12028–12032

Nakajima M, Welch D, Belloni PN and Nicholson GL (1987) Degradation of basement membrane type IV collagen and lung subendothelial matrix by rat mammary adenocarcinoma cell clones of differing metastatic potentials. *Cancer Res* **47**: 4869–4876

Reich R, Thompson E, Iwamoto Y, Martin GR, Deason JR, Fuller GC and Miskin R (1988) Effects of inhibitors of plasminogen activator, serine proteinases and collagenase IV on the invasion of basement membranes by metastatic cells. *Cancer Res* **48**: 3307–3312

Schalkwijk J, Chang A, Janssen P, de Jongh GJ and Mier PD (1990) Skin derived antileukoproteinases (SKALPs): characterization of two new elastase inhibitors from psoriatic epidermis. *Br J Dermatol* **122**: 631–641

Schalkwijk J, de Roo C and de Jongh GJ (1991) Skin-derived antileukoproteinase (SKALP) an elastase inhibitor from human keratinocytes: purification and biochemical properties. *Biochim Biophys Acta* **1096**: 148–154

Schalkwijk J, van Vlijimen IMJJ, Alkemade JAC and de Jongh GJ (1993) Immunohistochemical localization of SKALP/elafin in psoriatic epidermis. *J Invest Dermatol* **100**: 390–393

The Japanese Society of Esophageal Diseases (1992) *The Guideline on Carcinoma of the Esophagus*. Kanehara: Tokyo.

Wooley DE (1984) Collagenolytic mechanisms in tumour cell invasion. *Cancer Metastasis Rev* **3**: 361–372

Wiedow O, Schroder J, Gregory H, Young JA and Christphers E (1990) Elafin: an elastase specific inhibitor of human skin. Purification characterization and complete amino acid sequence. *J Biol Chem* **265**: 14791–14795

Wiedow O, Lundemann J and Utecht B (1991) Elafin is a potent inhibitor of proteinase 3. *Biochem Biophys Res Commun* **174**: 6–10

Zou Z, Anisowicz A, Hendrix J, Thor A, Neveu M, Sheng S, Rafidi K, Sefter E and Sager R (1994) Maspin, a serpin with tumor-suppressing activity in human mammary epithelial cells. *Science* **263**: 526–529

© Cancer Research Campaign 1997